# Sporting Resilience During COVID-19: What Is the Nature of This Adversity and How Are Competitive Elite Athletes Adapting?

**DOI:** 10.3389/fpsyg.2021.611261

**Published:** 2021-03-03

**Authors:** Sahen Gupta, Paul Joseph McCarthy

**Affiliations:** Department School of Health & Life Sciences, Glasgow Caledonian University, Glasgow, United Kingdom

**Keywords:** COVID-19, sporting resilience, adaptation, elite sport, positive psychology, adversity

## Abstract

The COVID-19 pandemic is a global health issue which has severely disrupted and deferred several landmark international sporting competitions. Like the general population, athletes have faced direct psychological consequences from COVID-19 in addition to cancelation of events, loss of support, lack of training, loss of earnings, hypervigilance, and anxiety among others. The aim of the present research was to identify the adversity experiences of athletes caused by COVID-19 (study 1) and explore the process of resilience used by competitive elite athletes for positive adaptation (study 2). Research has indicated psychological resilience to be a protective factor against similar adversities in the sporting context. The study uses an across-cases qualitative design comparing the real-time lived experiences of athletes during COVID-19 using narrative analysis. Data were collected from 10 competitive elite athletes from various countries, as part of a larger doctoral dissertation study during the lockdown period, using in-depth experiential interviews. Study 1 presents detailed narratives on the loss and incongruence, which were the two major adversities experienced. Study 2 outlines the process of resilience as narrated by the participants through the emergent and minimal-impact resilience trajectories. We discuss recommendations for interventions and the role of sports psychologists, coaches, and sporting organizations in ensuring athletes’ mental health and their rehabilitation into post-COVID sports life.

## Introduction

The COVID-19 pandemic, classified as a global health emergency, is the most serious disease outbreak since the 1918 H1N1 influenza pandemic emergency (Casadevall and Pirofski, 2020). The confirmed cases globally stand at over 83 million, with over 1.8 million COVID-19-related deaths on January 5, 2021 (World Health Organization, 2021). The pandemic has caused industry closure, changed daily life patterns, caused financial loss and unemployment, and disrupted social and community support, all of which have a clear psychological impact (Ho et al., 2020). Preliminary findings indicate that social isolation and lockdowns equated to spending 20 + h per day at home, causing chronic exposure to stress, self-protective behaviors, elevated anxiety levels, and depressive symptoms (Altena et al., 2020; Li et al., 2020; Wang et al., 2020). Shear (2012) noted how closure and grief processes have been suspended in the face of rupture, interruption, and loss of daily routine, death-triggering anger, helplessness, guilt, and shame. International tournaments such as Tokyo 2020 Olympic Games and UEFA Euro 2020 scheduled for multiple cities were postponed/canceled since travel is a major mode of transmission (Tian et al., 2020; Widdop et al., 2020). Health and safety considerations, intensified media scrutiny, and public perceptions have postponed/altered the format of sporting events (Ippolito et al., 2020; McCloskey et al., 2020). The substantial impact of COVID-19 on all levels of sport has been mediated through these rapid and irregular developments (Begović, 2020; Fullagar, 2020; Parnell et al., 2020).

Sporting organizations and its many stakeholders, most notably athletes and coaches, are faced with immediate impacts, i.e., health/infection, and longer-term threats, i.e., funding/talent development (Ngaluafe, 2020). There have been calls for thorough examinations of the influence of COVID-19-related changes in elite sport (Parnell et al., 2020). Long-term changes post-pandemic to redesign sport culture for greater accessibility, inclusivity, and safety have also been made (Leberman et al., 2020). Doherty et al. (2020) suggest a need for “leaning on evidence in turbulent times” while charting out directives for return to sport (p. 2). Schinke et al. (2020a) have cross-culturally evidenced the impact on high-performance athletes across three stages: before Olympic postponement, during Olympic postponement, and reset toward Tokyo 2021. Even before formal postponement, athlete life plans are in flux, causing stress responses. After postponement, athletes have had constructive and debilitating responses which range from intrinsic development to burnout syndrome, feelings of alienation, and poor coping compounded by social isolation (Schinke et al., 2020a,b). Similar evidence has also emerged from Japan (Taku and Arai, 2020) and Spain (Clemente-Suárez et al., 2020). Social isolation, conceptualized as an abrupt diversion in an athlete’s career, could also trigger dissociations from athlete identity and negatively impact mental health (Brewer et al., 1993, 2010). However, evidence-based psychological support is crucial because student-athletes who received more social support reported better mental health and well-being (Graupensperger et al., 2020).

Building upon the preliminary evidence provided, the present study offers insight into the lived experience of the adversity and the process of resilience adaptation in athletes. Violations of newly imposed health policy by athletes (cf. BBC, 2020; Ramsay, 2020; Trenaman, 2020) have also raised questions on whether professional athletes are held to a higher standard with expectations on them to be role models (Leng and Phua, 2020). In community sport, interdisciplinary collaboration has been noted to be a response route to the complex problems presented by COVID-19 to build a more resilient future (Doherty et al., 2020; Timpka, 2020). For elite athletes, Schinke et al. (2020a) also note the relevance of resilience in times of COVID-19, noting that “as for Olympians, they are and will continue to be forged in (and through) adversities” (p. 272).

“Resilience,” as a word, has seen widespread use by media and government officials, owing to its layperson meaning. It has been used as a way to facilitate adaptation despite the COVID-19 adversity. We conceptualize COVID-10 and the consequent national lockdowns as an adversity since it is a “state of hardship or suffering associated with misfortune, trauma, distress, difficulty, or a tragic event” (Jackson et al., 2007, p. 3) and distinguish it from stressor and trauma as per clarifications posited by Howells et al. (2017). This conceptualization of adversity also fits in within the theoretical position, viewing the COVID-19 pandemic as a change event in a sporting career which is distressing, derailing, and difficult. Psychological resilience is “the role of mental processes and behavior in promoting personal assets and protecting an individual from the potential negative effect of stressors” (Fletcher and Sarkar, 2012, p. 675). Contextualized to the sports context, sporting resilience is “the environmentally adaptable, interaction dominant, dynamic-process trajectory that encompasses a sporting individual’s metacognitive–emotional–behavioral capacities to maintain a positive equilibrium and successfully adapt to a diverse range of sport-related adversities” (Gupta and McCarthy, in press). Academia has provided a more nuanced approach to how resilience is used/can be used in response to the disruption caused by COVID-19 (cf. Barton et al., 2020; Polizzi et al., 2020). Bonanno (2020) negated the equating of resilience to a panacea, stating “that no single factor determines resilience for a population” (p. 1).

Although literature has provided commentaries, theoretical frames, and perspectives, there is limited empirical evidence to lean on to understand resilience during COVID-19. However, the model of uncertainty distress tailored to COVID-19 psychosocial reality (Freeston et al., 2020) provides insight into the impact of adversities and adversities which are simultaneous. Resilience is a temporally stable but dynamic psychosocial process that unfolds over time (Fletcher and Sarkar, 2012; Bonanno and Diminich, 2013; Galli and Gonzalez, 2015; Bryan et al., 2017; Hill et al., 2018). Many of the established protective factors of resilience such as perceived/tangible social support (Fletcher and Sarkar, 2012; Morgan et al., 2013, 2015; Brown et al., 2015; Codonhato et al., 2018), sense of meaning/belonging (Hall, 2011; Meggs et al., 2016), and motivational climate (Machida et al., 2013; Chacón-Cuberos et al., 2019) have been severely disrupted. Athletes either cannot access them or do not have the quality of protective resources they used to. For example, mastery and a sense of control enable a resilient response in the face of adversity (cf. Galli and Vealey, 2008; Fletcher and Sarkar, 2012, 2016; Morgan et al., 2013), which has been severely compromised because of the COVID-19 pandemic. Howells et al. (2020) have also noted the cultural script prevalent in how societies should respond to adversity. However, it is beyond the scope of the study to operationalize post-adversity growth since athletes were engaged in resilience response since COVID-19 lockdowns were ongoing.

Most athletes are still engaged in their resilience process since the threat of the COVID-19 adversity to physical and mental health remains. This complexity of acute chronicity of the adversity and the non-normative psychosocial circumstances provides the rationale for an examination of the adversities faced and the process of resilience engaged in by athletes through their lived experiences in this study. This research will adopt a narrative analysis to capture the experience of COVID-19 adversities and the resilience process that competitive athletes engaged in. Narrative analysis is an emerging research approach in sport psychology and will locate the dimensions of stories to best answer the research question (Smith and Sparkes, 2009; Smith, 2010). This technique peers into and examines the athlete’s lived phenomenological experiences, allowing for exploration of the actions over time (Carless and Douglas, 2013; Smith, 2016). To best answer the research question, we divide this research into two studies. Study 1 explores the nature of adversities faced; study 2 explores the complexity of the process of resilient adaptation. Studies 1 and 2 are conjoined under the philosophical stance of narrative constructionism (Sparkes and Smith, 2008; Esin et al., 2014) but use different narrative techniques to answer two specific avenues of the research question, converging into a holistic discussion. The findings of the study provide insights into the lived phenomenological experience of adversity and resilience specifically linked to COVID-19. This provides applied practitioners with an insight into the lived experience, helping to guide one-on-one therapeutic interventions and how to promote psychologically informed online training and psychological support and guiding other support staff.

### Research Question

This study aimed to explore the nature of adversities faced by competitive elite athletes during the global COVID-19 lockdowns and to understand the complexity of the process of resilient adaptation.

## Method Overall

### Research Paradigm and Methodological Congruence

Morse (1997, 1999) and Holt and Tamminen (2010) recommend “methodological coherence” (p. 419), which this study strived for by establishing consistency through ideation, research question, philosophical orientation, and theoretical perspective (Mayan, 2009). This study adopts a stance of ontological relativism (Creswell, 2013) and sits in an interpretivist–realist epistemology, concerned with understanding the reality of COVID-19 and resilience as engaged subjectively by athletes. The focus of analysis is on the unfolding of resilience in the lived experience of competitive elite athletes during COVID-19 lockdowns through their narratives. The hermeneutical differences and lived experiences of athletes were prized to secure authenticity to understand the nature of adversity and complexity of resilient adaptation during COVID-19 lockdowns. Narrative analysis was chosen to secure a detailed understanding at the individual level while retaining ecological validity since individual narratives are personal to them, but the structures that shaped them such as the lockdowns are not theirs alone (Riessman, 2008).

### Recruitment

Purposive sampling was undertaken using the formalized classification of athletes (see Swann et al., 2015). Per the classification, competitive elite athletes are athletes who compete at the international level regularly and have had success. Participants who were over 18 years and in countries with lockdowns were recruited. Both team and individual/team sports were included to provide a holistic idea about the nature of the adversity and the process of resilience in a time of isolation since there could be potential differences as per existing theory (Morgan et al., 2015, 2019). Competitive elite athletes were approached since they were likely to have resilient characteristics that enabled them to withstand stressors for achieving success in their sport (Hardy et al., 1996; Krane and Williams, 2006; Fletcher and Sarkar, 2012).

Potential participants were informed about the background of the study upon first contact. Phase I screening was conducted using the Brief Resilience Scale (Smith et al., 2008), in line with recommendations from Windle (2011) regarding its satisfactory reliability and validity. Participants self-reporting their resilience above normal threshold (i.e., 3) were invited for phase II interviews. Institutional Ethics Committee clearance was obtained prior to commencement. The participants were briefed on the aims, procedure, and outcomes, following which informed consent was obtained. The participants were told to inform the interviewer if they felt any discomfort in discussing COVID adversity due to its ongoing nature, in which case the interview would be terminated immediately without prejudice. The interviewee was a trainee psychologist and would suggest referrals to the participants should they require psychological support through this time.

### Participants

In phase I, 15 competitive elite athletes (*M*_*age*_ = 26.33, SD = 7.96; male/female = 7/8) filled in the Brief Resilience Form (Smith et al., 2008). All athletes screened at phase I self-reported above-average resilience but were not informed about their psychometric scores until after the interview to reduce bias. Equal numbers of male and female athletes were chosen from countries around the world for representative results. Although attempts were made to secure a globally representative sample, major sporting nations such as the United States, Brazil, and Russia were excluded since they did not have an extended period of complete societal lockdown. All participants were contacted and invited for the interview. In phase II, 10 participants of equal gender representation participated in the interview (*M*_*age*_ = 26.10, SD = 6.41). Sample demographic details are illustrated in Table 1. The authors recognize a Eurocentric trend in the sample but highlight the narrative voices of competitive elite athletes from other societies. Although there are no fixed rubrics on sample size (Vasileiou et al., 2018), this study’s sample was limited by pragmatic concerns such as lockdown, ending in unanswered forms and participants not responding to interview invites.

**TABLE 1:** Participant demographic information.

Pseudonym	Age/gender	Participating sport	Representation	Experience
Frisbee	21/male	Frisbee	Australia	7 years
Natasha	26/female	Rugby	Scotland	5 years
Jack	24/male	Badminton	Team GB	20 years
Julia	30/female	Lacrosse	Scotland	17 years
Maria	19/female	Tennis	Scotland and Team GB	14 years
Bruce	22/male	Canoe Slalom	Ireland	9 years
Veronica	31/female	Netball and basketball	Gibraltar	23 years
Tessa	28/female	Curling	Team GB	17 years
Alex	40/male	Savant	Team GB	20 years
Rohan	42/male	Golf	India	34 years

### Procedure

The screened participants were invited for an online interview and were informed that the interviews would take approximately 45 m to 1 h of their time with no payment/incentive provided for participation. Microsoft Teams was used for a confidential and secure encrypted connection. In line with ethical research, to ensure participant confidentiality, anonymity, and safety (participant and researcher), the online interviews took place in a private room. Following written and verbal consent, interviews were conducted and audio-recorded with a Dictaphone. After the interviews, data were anonymized, transcribed, and encrypted with AESCrypt. The researchers had sole access to data in line with general data protection regulation (Voigt and von dem Bussche, 2017), the Data Protection Act (2018) regulations, and the BPS-GDPR guidance for researchers (BPS, 2018). The first author conducted verbatim transcription of data. The transcripts were anonymized and provided with a participant ID and code name linked to their sport, balancing the protection of participant identity and data integrity (Saunders et al., 2015).

Data were collected using a semi-structured interview schedule, with probes and questions offering the space to discuss the experience of COVID-19-related adversity and how they engaged their resilience. The interview questions were open-ended, and the participants were told to be conversational. For example, “So you mentioned quarantine, which is the elephant in the room. How has that experience impacted you?” The interviewer encouraged all the participants to tell their own stories; however, they wanted to and took on the role of an “active listener” during the interview (Smith and Sparkes, 2005). Techniques from experiential interview scheme, such as reflecting on the lived situation, were implemented (Janz, 1982). Life story interview techniques were used to document the adversity experience over a period of time (Atkinson, 2007). This allowed exploration of adversity experiences and the process of resilient adaptation through the participants’ narratives. It was reiterated that there was no obligation to share narratives and that the participants could withdraw at any point. At the time of the interview, all participants were residing in countries with complete national lockdown.

### Methodological Rigor

Methodological rigor was established by incorporating authentication strategies such as peer debriefing during the research process to ensure credibility, transferability, dependability, and confirmability (cf. Morse et al., 2002). Coding was done initially by the first author and independently checked by the second author and another critic friend. The results which emerged were critically debated to ensure consistency in the framing of the lived experience through the narrative analysis. Quality control was adjudicated using what Sparkes and Smith (2014) term time-and-place contingent characteristics. The results were judged *via post hoc* evaluation (cf. Fletcher and Sarkar, 2012) by using quality criteria of contribution (i.e., a worthy topic of inquiry, credibility, and resonance) (Tracy, 2010). To briefly explain, we feel that our research is a timely and essential contribution since sport psychology is still figuring out the impact of COVID-19 on the psyche of everyone involved in sport. As such, the “practical level of truth” (Giacobbi et al., 2005, p. 22) uncovered by this study holds relevance for policy development, applied sport psychology practice, and coaches since it provides an insight into lived experience. Credibility was also ensured through triangulation, where interview transcripts and interpretations were checked with the participants where possible, which ensured that the rigor and data analysis were not biased. Finally, we hope that providing descriptive quotes in the article illuminates the narratives to the readers and resonates with the experience of all in the sporting context who are affected by COVID-19.

## Study 1: Method

### Data Analysis

Data were subjected to a narrative thematic analysis (Riessman, 2008). Specifically, the researchers read and re-read each transcript for familiarization and noted initial thoughts. Both the location of telling (subjective context) and the location of the story (macro context) were noted since the contexts shape the narrative and need to be explicitly considered in the analysis (cf. van Vuuren and Westerhof, 2015; Levitt et al., 2017). In line with recommendations, the focus was on what is said rather than for what purpose or how or to whom (Riessman, 2008). Blocks of text which represented a thought or meaningful point related to adversity experience were identified and labeled as a raw-data theme. Since the goal of thematic narrative analysis is to tell the “whole story” while preserving the sequencing of events, the themes reflect temporal elements of the experience of adversity caused by COVID-19 lockdowns. The themes noted below were reflected in all the participant narratives. Themes were compared and contrasted with each other and finally represented in a rich and layered narrative detail from multiple participant quotes.

## Study 1: Results and Discussion

The results are framed under two narrative themes which explored the nature of adversities faced by competitive elite athletes during the global COVID-19 lockdown. The themes outlined are idiosyncratic and ecologically valid since they were found universally across the narratives of all participants. The components of the narrative are in equal parts lived experiences during the lockdown and were caused by it.

### Loss: Sport, Support, and Identity

Loss was a recurring part of the adversities faced by the athletes during COVID-19 lockdown. “Jack” summarized this succinctly when he exasperatedly stated, “I have *never* had 8 weeks where I have not stepped on a badminton court; so, it’s weird.” This feeling of losing a sense of normality is associated with the loss of their sport (i.e., ability to engage in sport, train, and compete was echoed by all participants). The narrative also encompassed adverse emotional reactions to this loss. As “Frisbee” recounts:

“For example, yesterday, I was waiting 3 days for a 10-min walk, which is what I get, and then I was told that I would be lucky to get a walk in 6 days and I would most likely not get a walk at all till I am out of here and in that moment I got really annoyed, put the mattress against the wall and started punching it. And I was in a really foul mood for about half an hour. And then I sat down why I was… like what was the switch, like I had an expectation that dropped, that could not happen, and I was getting annoyed and really upset and getting frustrated. I guess being cooped up is difficult for us athletes, more so than normal people, because being out and physical is what we do.”

“Jack” related his situation to this line of thought as well reflecting on his experience through the narrative of what all athletes must be going through, “Quite a lot obviously, for athletes, this part of time is quite difficult because obviously we can’t play, so I’ve gone from playing… like I have never had more than 2 weeks off from badminton, maybe 4 weeks due to injury or something like that.”

“Bruce” expressed frustration while noting how the pandemic and lockdowns have impacted training and his routine, “We have got a white-water course in ^*******^ which is basically why I moved there, but that has been closed since well forever, well 9 weeks.” He also discussed how this has made sport and “training on my own less enjoyable,” a sentiment also expressed by all other participants.

Loss of sport and training was a major focus in the narrative of “Veronica.” Painting a detailed picture, she noted:

“We, like myself and other competitive athletes, I am used to training, every day, except for one… so we train 6 days a week. We have one rest day…. Most of the time, it is on court…. Because whether we’re doing conditioning or whether we’re doing tactics or when we’re just getting together to see some video analysis… like everything is done with your teammates and in an environment where you have the coach there… and now. We, we cannot be in an area… like the netball court… you cannot just go down and play… this has been a major difficulty…And it’s all specific, you know, to the sport, whereas now we haven’t had the opportunity because we cannot be in contact with other people… Training is usually 2 h long every day. And now I have nothing to do…. So, that’s had a big, knock-on effect on our fitness… So, we have had to try and replicate as much as, as much as we can, but obviously honestly it’s not good enough.”

Loss of sport and training also had a tangible effect on the mental health and psychological side of sport, which was also lost. “Alex” noted how the “psychological environment is important… if you’re around people who are negative then it’s a factor, and now there are a lot of people who are understandably negative.” The performance-oriented side of psychological preparation was also severely hampered with the loss of sport. “Tessa” notes: “But the thing I’ve struggled with is not being able to go to the gym…. And I think I spoke earlier about having goals… right now, it is almost impossible to. My problem is I don’t really have any goals at the moment.” The narrative of “Julia” also highlighted similar adversity themes as she explained how “I have tried to, like, stay fit. I am doing, like, strength sessions and conditioning sessions still, but maybe not as many as I would be doing, like, normally, and there are some things that I just cannot cope with, like I just can’t cope with even thinking about the testing so I have just completely ignored that… like, for example, I used to have, like, this fear of God, of missing the testing, or something like that, but I just have not done any of the testing, and for me, you know, work has also been very challenging and stressful… so just yeah… things in my mind.”

Another major loss experienced by athletes has been the loss of support and “having a good sport system” (i.e., sport support systems). Linked closely to loss of sport as shown above, “Jack” noted how:

“For us, like, full-time professional athletes, we have got the coaches, the physios, the psychologists, and the sport performance lifestyle people. Like, having these people around, people you can, like, chat to, and have, like, honest conversations with, I think what the environment and the sport staff having a good environment rubs off on you and the players that you are training with… and it’s good to have that different areas of expertise, and this COVID-19 thing has prevented that.”

This major adversity has led to other difficulties for the athletes such as skill acquisition and talent development. “Frisbee” narrates a recent experience, “There is something I have been trying to do during quarantine, its fluid movement, so handstands, rolling into flips and what not, and I have tried to do it by myself, and it get stuck …And without someone showing me, I feel like I would not have to get anywhere.” “Veronica” also narrates how loss of support (technical, emotional, social) has also forced other forms of innovation during lockdown:

“We’ve had to adapt to different ways of training, how to do home workouts. You’ve had to be following and doing, like, fitness, personal trainers online… We’ve just had to adapt the way we’ve done ball skills…like, doing it individually or with a family member… which may not be as experienced… Because you have to try to teach them how to do the things you want them to do. And then the fitness, the fitness is not the same at all. like, home workouts we are doing for 1 h or 45 min.”

“Bruce” also noted that losing the direct support offered by coaches and others in the sport system has had adverse effects as well, “’cause quite a lot of the things that athletes and coaches are struggling with at the cause if they have guided an athlete the whole way throughout their career, now they can’t because of the restrictions… and the athlete is sort of lost.”

Although not explicitly stated, the participants indicated a loss of identity caused by the prevailing psychosocial conditions. This is seen in the dialectic descriptions of the way the participants struggle to describe self away from the dominant sport-culture performance narrative despite no actual sport participation and competition (Douglas and Carless, 2006). Interestingly, all participants followed this pattern identification of athlete role and hinted at a sense of reduced accomplishment due to the COVID-19 reality and the corresponding loss of cognitive and social utilizations of the athletic identity (Brewer et al., 1993). However, this evidence is not an inference on whether athletic identity is a Herculean muscle or an Achilles heel (cf. Brewer et al., 1993) but an acknowledgment that there is a sense of loss of a major aspect of self-identity for competitive elite athletes. Although there is no direct indication to this loss, this acknowledgment secures its rationale because narratives are the “sea of stories that seeps into our consciousness and our very identity” (Murray, 2003, p. 98). Therefore, its implied presentation by the participants can be taken as preliminary evidence of its operation in their phenomenological reality.

### Incongruence

Analysis also confirmed incongruence in the narrative presented by the athletes. This incongruence represented a form of dissonance between the usual structured, goal-directed environment they are acclimatized to which the relatively aimless COVID-19 isolation situation brought. This incongruence manifested in feelings of impotence because of the psychosocial reality *versus* a need for mastery which elite athletes are acclimatized to. This incongruence has surged into psychological distress, ruminations, negative emotions, and loss of motivation, constituting a major adversity. As “Julia” narrates in relation to collaborating with rugby players for training:

“I just can’t add anything like that to my mind right now so I have kind of ignored and muted all those messages right now… so yeah, I’m not, like, I think I have just tried to protect myself from stress, so I don’t know if that is knowing my limits and, like, making a decision, like, I know I can’t cope with that, and I don’t know if that’s me not being resilient, I’m not sure…”

All participants also noted a corresponding impact of this incongruence on their athletic identity general worldview. “Tessa” recounts how the incongruence and unstable COVID-19 situation has led to “So I really struggled with aiming toward something…. And I got a bit out of the habit of doing or giving my full effort to some of my workouts because I really was struggling my motivation, because I kind of felt *what’s the point*?”

“Jack” also pointed to several factors of the situation as leading to this incongruence, which better illustrates the nature of adversities faced by competitive elite athletes, illustrating that:

“It’s hard to … it’s hard to motivate yourself when you feel like there is nothing to work toward ‘cause you don’t know if tournaments are gonna happen again, and when we are going to get back to training, like, it’s so up in the air, so it’s really hard, and you really have to be resilient because you cannot focus on looking at the longer-term goals… and maybe… 3 months from now we could be back on a court and being focused enough to make that our goal, and we need to be in a good shape when that happens and it comes to that…”

“Maria” also noted the negative impact of losing control over sport as losing control over her coping, stating that “I have also been going through some personal things off the court, with boyfriends and things like that, uhm… so I have really struggled actually this past week to try and draw upon my resilience…” The sense of impotence shared by the athletes is best captured in the narrative of “Tessa”:

“I think being an athlete is a bit of a roller coaster sometimes. Obviously, there’s going to be very high highs. But there’s always lows, and I think anyone can go through their career without having lows, and you’ll have multiple, multiple every year…. and we kept having lows this year didn’t we?”

Another aspect of this incongruence experienced by athletes was the struggle to be “perfect” athletes and normal “victim” human beings. This is reflected not only in the narrative throughout as the participants noted their struggles but also by the fact that they have to keep being athletes and do all that is expected of them. “Julia” narrates the difficulty of managing this incongruence:

“’Cause it’s been very hard, like, the mixed messages that are coming through is a major reason for that, some people are, like, this is a totally unprecedented time, you shouldn’t be pressured and things, but then people are also, like, if you’re not training, other people will still be training, and they will have come out of this having lost less fitness than you have… umm… and then we have got our strength and conditioning coach who keeps firing out programs for us to do and like testing and stuff.”

This incongruence is also reflected through the attempts made by the participants to continue being elite athletes despite the COVID-19 reality and losing access to all sporting facilities. “Veronica” describes what it has been like:

“So it’s been a lot of frustration because, like, you’re, you’re used to, like, training with teammates for the same level and ability of you and you’re doing movements they know everything…. You don’t have to go through it, right? like, if you’re dealing with a family member, they might not even be able to pass or catch as well or know your movements… so it’s very frustrating to try and keep your training up…”

All the participants were frank and discussed feeling the need to live up to ideal perception of what an elite *should* be and their struggles with it. “Perfect” and “should” came up multiple times in every participant’s narrative. “Maria” noted that, at times, she has tried to maintain a routine and “control the controllable, so I trying to control the things that are in my control and all the little things that I do, I can’t say I am a perfect example because I have definitely struggled as well ‘cause it has not been easy not having those distractions in your life, like, playing, that is my passion.” Despite the far-reaching nature of this incongruence, a majority of the participants explicitly displayed a tendency reframing and undergoing challenge appraisal (Fletcher and Sarkar, 2012). “Natasha” framed this eloquently by describing how “I have been going, like, things are changing, we are all learning and we are all on the same page and it’s a bit of a rubbish situation, just let’s get on with it.” “Tessa” also echoes this sentiment, saying:

“I’ll be honest I’ve struggled completely honest many times… so we have been switched to, we’re quite fortunate because the curling season had finished, our competition season had finished…. And, yes, we missed some training, but the on-ice training, we didn’t miss … it is the best part of the season to miss ‘cause it’s after the championship.”

“Rohan” also looked beyond onto the larger psychosocial reality that he was a part of by stating: “been a bad effect not only on sports but also on the economy and many other things.” It is logically postulated that the process of reframing the adversity faced and engaging in challenge appraisal by all participants to mitigate the psychological impact of COVID-19 is because all participants self-reported high resilience scores. It needs to be highlighted that the themes of loss and incongruence prominently featured in the narrative are not mutually exclusive but rather two sides of the same coin of the COVID-19 psychosocial reality for competitive elite athletes.

## Study 2: Method

### Data Analysis

Narrative structural–quest analysis was chosen since resilience unfolds primarily at the individual level and can be traced much like a character arc in a narrative. It is suitable since it focuses on how the resilient adaptation narrative functions rather than the context of the analysis, i.e., COVID-19 adversity which has been explored in depth by study 1. Riessman (2008) outlines the six parts of a narrative structure: abstract, introduction, complication, evaluation, resolution, and coda. This congruently fits with adopted theoretical perspective of resilience (cf. Bonanno and Diminich, 2013; Gupta and McCarthy, in press) and provides study 2 with methodological coherence. The quest narrative has a plot structure story in which the person meets suffering head on, accepts the disability, seeks to use it, and commits by developing uncertainly (Smith and Sparkes, 2009). This is like the structure of resilience adaptation outlined in theory, i.e., the individual meets adversity which is major and exceeds available resources, which causes a disruption, then a depletion, followed by a metacognitive learning process, and finally a rebound with new learning and resources into positive adaptation (cf. Bonanno and Diminich, 2013; Gupta and McCarthy, in press).

The structural analysis teases out the resilient narrative of the individuals and helps understand the thoughts, actions, hopes, emotions, and psychological complexity (Smith and Sparkes, 2005, 2008). The focus of analysis was not on the performance narrative (Douglas and Carless, 2006) but on the resilience narrative as it was unfolding for positive adaptation during COVID-19 adversities.

## Study 2: Results and Discussion

**FIGURE 1:**
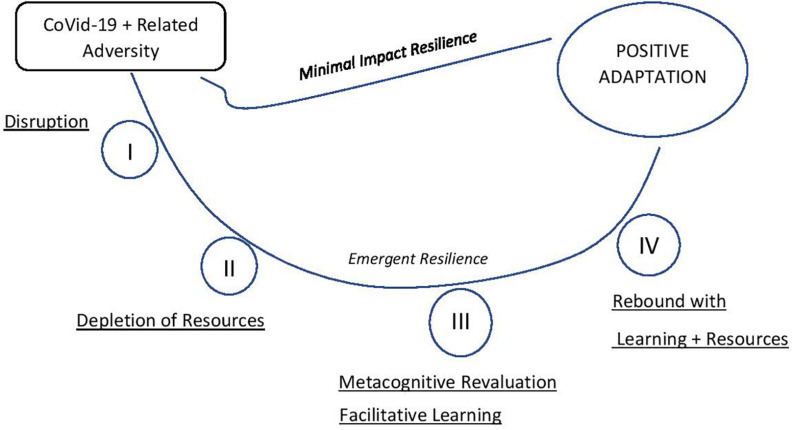
Model of resilience trajectories adapted to COVID-19 (adapted from Bonanno and Diminich, 2013; Gupta and McCarthy, in press).

Analysis of data was built upon the findings of study 1, which explored the tangible COVID-19 adversities in the lived experience of the athletes. Narrative quest analysis of the data is framed in extant theory (cf. Bonanno and Diminich, 2013; Gupta and McCarthy, in press) to best report the complexity of the process of resilience and positive adaptation undertaken by athletes (see Figure 1). The results were synthesized from a detailed analysis of specific individual narratives, each of which was interestingly found to have a high degree of similarity in their plot, suggesting a relative cross-cultural uniformity in the resilience process. In line with recommendations, whole narrative quests are not framed in theory; rather, a complete account of everything the participants conveyed is provided to provide an informative process description through lived experiences (Wong and Breheny, 2018).

After COVID-19, the participants experience a wide range of sporting and non-sporting adversities (see study 1) and drew upon their resilience. “Natasha” placed great emphasis and notes: “Oh yea, *totally*.”

All participants engaged in a positive reframing and challenge appraisal which signaled the start of their facilitative resilience response (Fletcher and Sarkar, 2012). Bonanno and Diminich (2013) theorized two potential pathways depending on the magnitude and relevance of the adversity in question. Because of the acute, unprecedented nature of the COVID-19 adversity, no participant reported progression of resilience response along the “minimal impact resilience” pathway (p. 380). The novel nature of the pandemic and the global lockdown were not previously experienced or even heard of by the participants. Therefore, identified resources could not be used to engage in a resilience response and maintain well-being throughout this extended adversity.

All the participants reported engaging in the “emergent resilience” process trajectory (Bonanno and Diminich, 2013, p. 379). The resilience responses progressed along stages but often oscillated back and forth based on time and situational factors in their psychosocial reality (Gupta and McCarthy, in press). Stage I—“disruption” was a recurring feature in the narrative, linked to the loss brought about by the adversity (see study 1). “Natasha” notes how “It’s been such a fast-evolving thing and everything is changing every shift you are coming in at and you have just got to go with it since you can’t not go with it… and I have been going like things are changing, we are all learning and we are all on the same page and it’s a bit of a rubbish situation, just let’s get on with it…”

“Rohan” reiterates this view by adding: “We are all through a bad time… sport has stopped… and that has been unusual… I am so used to getting up early and going to the course, but not now.”

However, all the participants’ narratives reflected an acceptance of the reality and a corresponding resilience response as they moved into stage 2—“depletion of resources.” The nature of adversity itself included loss of several components and resources as well (see study 1). “Alex” explicitly recounted how “Coronavirus says to me, ‘training in person isn’t going to work,’ what do you do?”

“Natasha” noted:

“We (team) have kept in contact even though we can’t train, we are communicating, but it’s a very real loss even though we are in isolation we have been doing fitness challenges, and that’s been really motivational not just to keep moving but also the realization that this is not forever; one day we will be back to play together again.”

“Jack” experienced an added adversity as he injured his knee running in lockdown. His narrative prominently featured the reverse oscillation of events which initiated another disruption–depletion process trajectory. He notes:

“For me, like, I got injured 2 weeks ago out running, I slipped and slid, like, a freak accident and I hurt my knee, and, like, it’s been tough to go from at least I was training and now I cannot really train ‘cause my knee is real sore, and it’s kind of been, like, this different side to my resilience that I have had to use again, and, like, I picked myself up and had to go through rehab, go through not being able to train, go through like a walk everyday.”

This acceptance of the unnatural reality and the various adversities it presented allowed all participants to engage in a metacognitive challenge appraisal and learning. This metacognitive appraisal included looking beyond individual adversity and looking at the larger world since the COVID-19 pandemic is a planetary phenomenon. This indicates a resilient response since adversity anxiety usually prompts rumination and tunnel vision thinking (Brooks et al., 2017). “Rohan” eloquently describes:

“We are all through a bad time… and my resilience has allowed me to have that positive perspective to think that it’s not just me, it’s the whole world who is facing this… and I know that, over the next few months, we will recover from this, and there has been a bad effect not only on sports but also on the economy and many other things… and if we think into the negative we will go deeper into the negative and things are getting better as well… if you see that medicines are coming out and all of that, so yeah… things are slowly opening and all of that… but yes.”

“Rugby” also highlighted the importance of learning to use social support in an innovative manner to overcome the various adversities faced, noting how her past sporting experience has been a major facilitative factor:

“I work in a team at work (as a nurse), so you know keeping the positivity and feedback since it’s been such a fast-evolving thing and everything is changing…every shift you are coming in at and you have just got to go with it since you can’t not go with it… and I have been going like things are changing, we are all learning and we are all on the same page and it’s a bit of a rubbish situation, just let’s get on with it… I think if I hadn’t played sports before I would have found it very challenging, the constant change, the fast-paced environment, not knowing what was going on… like…. And keeping the positivity, ‘cause there is a lot of stuff that I take from the team that I am in and the people that I play with into work and taken my attitude and being the positive person into work and kept that going…. Em. yeah… I don’t think I’d be okay if I did not have that team sports to fall back on and through this time as well we have kept in contact, even though we can’t train, we are all on WhatsApp groups, we are all communicating, we are building each other up, and have the realization that this is not forever; one day, we will be back to play together again…”

“Rohan” also states how learning and taking action and gaining a sense of mastery in the pandemic times was indicative of his resilience:

“See, I have interacted with many golfers and other sport players as well, but whenever I have talked to them, and even among normal people involved in sport and from my own experience, I think that without sporting resilience you can’t survive in any sport, and in times like these, I have used it a lot to, you know, try to be in control and stay ahead.”

“Veronica” confirms this, stating that:

“The whole journey you take…I think it builds character and personality and it makes you stronger mentally and physically…because if you’re thinking about yourself, like, not achieving certain results. You obviously only see, like, think of yourself and go ‘I need to, I need to make myself better’, and then have a positive mindset, or develop positive habits within exercising or eating or anything you’re doing… so…. I think having good resilience will make you a better athlete because, if you don’t have that, you’re gonna have…. You’re gonna put yourself down in a lot of the times where you could still achieve…. So, I think it definitely builds personality and experience, will help a lot to build resilience in you, but yeah, I think it had a positive effect to help me get through this.”

“Jack” illustrates a shift in perception as a major metacognitive re-evaluation and learning process which has directly led to his resilience response. He narrates:

“I feel like we are all going to come out of this with different perspectives on how much we rely on the sport to happen and on our sport to keep ourselves going. I think it’s gonna be a good thing by the end of it, but it’s gonna be hard work between now and then… Yeah, I feel like I could have easily been, like, really pissed off and angry that I had this accident (injury) and then because of it I cannot train; it would have been really easy to completely go off the rails!. cause I can’t train and do anything but I am actually a lot relaxed about it, to be honest, and am thinking of it like it’s an accident and how these things sometimes happen, and maybe 5 years ago I would have been really pissed off about it… but now, it is kind of, like, it happens, nothing I can do about it. I just need to focus on getting back to full fitness; so, in that way, it’s been good ‘cause it’s given an almost refocus because of this, and it’s much easier for me to do my training, do my rehab, simply because I have a focus, and it’s no longer just about maintaining fitness level till we are back on the court, but now it’s like I am down a bit, and I really need to work back up, and it’s all sort of added up to become a good thing, I want to say, cause “I feel like I had absolutely improved to refocus to training and stuff; it’s not ideal but there is nothing coming up that I am really needing to deal with, but rather like it’s another step on the road really.”

## General Discussion

The general discussion infers findings through theory and lived observation/practice experience to help the readers glean the maximum possible information for research and applied practice to contribute to the development of supportive, inclusive, and adaptive practices (Ryba and Wright, 2010). Competitive elite athletes regularly experience acute stressors of various forms (see, for a review, Sarkar and Fletcher, 2014) and often have a strong athletic identity and sense of self derived from participation in sport (Sparkes, 1998). Upon retirement and the removal of the sporting platform, athletes lose access to resources, support networks, and their sport, which leads to coping difficulties and mental health threats (Jewett et al., 2019). Findings suggest that the COVID-19 pandemic and the lockdowns have ensured that athletes experience an obligatory hiatus that is very similar in onset, experience, and reality to a forced retirement (Park et al., 2013; Jewett et al., 2019). Therefore, supporting athletes using career transition support and/or chronic injury psychological rehabilitation literature is one potential intervention.

Study 1 indicates that there are tangible losses and a sense of incongruence felt by competitive elite athletes because of the COVID-19 lockdown and pandemic. Clinical psychology research has provided evidence that prospection (i.e., simulation of future events allows individuals to generate embodied predictions of the emotional effects of events before the occurrence of the event) (Gilbert and Wilson, 2007). Findings provide preliminary evidence that the resilience responses to COVID-19 adversity by competitive elite athletes follow the risk-as-feeling hypothesis (Loewenstein et al., 2001). Anticipatory emotions and consequences lead to choices and behaviors that differ from the usually “optimal” behavior expected of elite athletes. Athletes have an active incongruence between being a “perfect athlete *vs* normal victim,” which serves to maximize personal psychological protectiveness and minimize harm.

Lack of cure/vaccines and no population immunity have increased feelings of danger and uncertainty in this health crisis. This uncertainty, combined with a lack of control, has become a source of psychophysiological variations of sympathetic activation, releasing unstable levels of catecholamines and cortisol. This further predisposes psychopathologies, such as anxiety, heightened stress response, and depression (Clemente-Suárez et al., 2020). The current COVID-19 crisis has uncertainty, unpredictability, and uncontrollability, all of which have been linked to anxiety (Grupe and Nitschke, 2013). The adversity experienced and the consequent resilient response by competitive elite athletes can thus be classified as non-normative. It is non-normative for core reasons: First, there is no experience involved, which is a core component of generating a sense of mastery key to resilience (Galli and Vealey, 2008; Fletcher and Sarkar, 2012, 2016; Morgan et al., 2013; Zurita-Ortega et al., 2018). Second, many usual resources accessed to enable a resilient response such as perceived/tangible social support (Holt and Dunn, 2004; Mummery et al., 2004; Yi et al., 2005; Galli and Vealey, 2008; Hall, 2011; Fletcher and Sarkar, 2012; Morgan et al., 2013, 2015, 2019; Brown et al., 2015; Lu et al., 2016; Yamada et al., 2017), motivational climate (Machida et al., 2013; Codonhato et al., 2018; Chacón-Cuberos et al., 2019), facilitative environment (Fletcher and Sarkar, 2016; Galli, 2016; Wagstaff et al., 2016), and identity (Cowden and Meyer-Weitz, 2016) are lost. Loss of training and sport also constitutes a loss of an adaptive coping mechanism often used by athletes (Park, 2000). Resilient athletes diverged from the general adaptive tendency, which involves avoidance of situations involving threat uncertainty and feared potential averse emotional impact (Paulus and Stein, 2006; Lovibond et al., 2009; Grupe and Nitschke, 2013). They rather engaged with the adversity threat and sustained the disruption and depletion to engage in a metacognitive revaluation and learning process, i.e., the emergent resilience process trajectory (Bonanno and Diminich, 2013).

The findings appear congruent to the Model of Uncertainty Distress in the Context of Coronavirus recently proposed by Freeston et al. (2020). Athletes’ experiences of adversities and uncertainties were simultaneously actual and perceived, which has led to the complicated nature and consequences of the lived experience post-adversity (see study 1). The resilience responses undertaken with the emergent resilience pathway are one example of an uncertainty-reducing thought–action which has, to some extent, mediated the relationship between perceived uncertainty and the intolerance of uncertainty. However, we connect these preliminary notes cognizant of the fact that the resilience processes part of the participants’ narratives is still ongoing. The COVID-19 pandemic and its associated uncertainty and threats are still ongoing, ensuring constant anticipatory anxiety since there are no environmental safety signals reliably indicating the threat as over (Seligman et al., 1971; Butler and Mathews, 1987). The resilience of the athletes has played a role in “promoting personal assets and protecting an individual from the potential negative effect of stressors” (Fletcher and Sarkar, 2012, p. 675). However, resilience constitutes of “dynamic processes by which biopsychosocial system returns to the previous level of functioning following a perturbation caused by a stressor” (Hill et al., 2018, p. 367). Therefore, it would be precipitous to infer that the participants of the study have used their resilience for positive adaptation since the adversity they are to adapt to is still ongoing.

### Implications

The results provide narrative evidence support to the conceptual model of Samuel et al. (2020) of the coronavirus pandemic as a change event in a sporting career. Study 1, capturing adversity experiences, fits squarely within coronavirus stage A, and the findings of study 2 showing the complex process of resilient adaptation reflect coronavirus stage B (cf. Samuel et al., 2020). A helpful parable to understand the COVID-19 adversity and resilience is to liken it to other similar acute, long-term, restrictive adversities and uncertain, non-controlled change events. Faced with these unexpected changes, elite athletes have to considerably change their sport engagement (Samuel et al., 2015). A textbook example is a sudden long-term injury or other complex experiences. Both in COVID-19 and long-term injury, the endpoint to return to “normal” sport is unknown, but athletes still have to process and continue with their career preparation. COVID-19 is a novel psychosocial threat to health, life, and livelihood. One strategy for applied practitioners to support athletes is to engage them in a metacognitive challenge appraisal by drawing evidence from experiences of overcoming similar types of adversity in their sporting careers, for example, reflecting on strengths and experience of overcoming a chronic adversity such as injury and how those same capabilities can combat COVID-19 adversity.

This study holds relevance since it provides coaches, sports psychologists, and sporting organizations with the lived reality of adversity and resilience as experienced by athletes. It is not a general assumption, but it is a demonstration of the depth and breadth of the experience. This richness will help better understand and therefore better direct support. The insight into the resilience process that highly resilient athletes are engaging in demonstrates an emphasis on acceptance and constant relearning as key. We recommend that an applicable practice philosophy or intervention theoretical frame would be to move away from fixed mindset to a growth mindset which improves resilience (Brady and Alleyne, 2017). This would allow better resilient adaptation to new sport protocols such as quarantine, testing (MacInnes, 2020), and re-entering a changing financial market which might alter career trajectories (Transfer Market, 2020). We concur with Schinke et al. (2020b) who advocated the need to develop an organic approach (i.e., placing the focus on the person behind the athlete). This study gives the raw narrative experiences, allowing practitioners to get an understanding from the athletes’ perspective and guide intervention design with that cognizance.

We are cognizant of the fact that we conducted the study during uncompromising lockdowns, but we are now moving forward in changed circumstances. Sport and the athlete’s sporting careers are continuing on in different ways, such as lack of formalized training or rehabilitation support, bubbles, and/or closed-door competition. There is also a possibility of various forms of lockdown should outbreaks occur. Therefore, the results and the topics of the study hold importance as a navigational marker for sports psychologists, coaches, and sporting organizations. Should sport psychologists have to support clients who have returned to local lockdown because of outbreaks or a positive test, this study provides an idea of what athletes faced, giving them a perspective of their clients. Therefore, we recommend that psychological support should be focused on the maintenance of mental health and the development of psychological coping skills for self-care rather than performance indices. For work with adolescent and early collegiate athletes, introducing psychoeducational life skill training in addition to psychotherapeutic support is a holistic approach for intervention (cf models Hodge et al., 2013; Sudhesh et al., 2020). Another recommended intervention strategy is to facilitate the development of self-regulation behavior in athletes, which is an important protective factor of resilience among youth at risk of social exclusion and in sport (cf. Artuch-Garde et al., 2017; Gupta and Sudhesh, 2019). This would also promote a sense of control and environmental mastery, which promotes resilience (Sarkar and Fletcher, 2014). Practitioners should ensure digital upskills to be effective in the online modality of support while keeping service delivery considerations of accessibility, working alliance, confidentiality, disinhibition, verbal/non-verbal cues, time delay, and technological issues in mind (cf. further open-access reading, Price et al., 2020).

The findings constitute the lived experiences where the athletes are coming from and can serve as a map of the psychological terrain that the new-normal sport is heading toward. There has been a very real impact on the athletic lives because of the COVID-19 adversity, and athletes are still engaging in their individual resilience trajectories. This study shines a light on some aspects of the impact, but it is as yet unknown what its long-term impact on psychological fitness is. For instance, a limitation of the study is the collection of data at a single point of time rather than at multiple temporal points. We need further research to establish a timeline of COVID-19 adversity experiences.

## Conclusion

This article presents the nature of adversity experienced by competitive elite athletes during COVID-19 lockdowns (study 1) and the corresponding process of resilient adaptation (study 2). The dynamic and longitudinal nature suggests that, for the foreseeable future, athletes will continue to engage in their resilience process since the adversity is ongoing, not a bygone *ex post* fact of the past. This article uses a narrative technique to give a feel of the lived experience of athletes during an unprecedented time. Essentially, it is trying to explain what we did not know framed within the existing scientific theory that we know. The study is not a sweeping future prediction but an insight into the lived experiences, providing a thorough evidence of the psychological position athletes are coming from, to facilitate research and applied practice on where they go. The article is also a guide should this phenomenological reality of lockdown returns, although the authors sincerely hope that it does not.

## Data Availability Statement

The original contributions presented in the study are included in the article/supplementary material. Further inquiries can be directed to the corresponding author/s.

## Ethics Statement

The studies involving human participants were reviewed and approved by the PSWAHS Research Ethics Committee of Glasgow Caledonian University. The patients/participants provided their written informed consent to participate in this study.

## Author Contributions

Both authors were involved in the research design analysis and manuscript preparation. SG conducted the interviews.

## Conflict of Interest

The authors declare that the research was conducted in the absence of any commercial or financial relationships that could be construed as a potential conflict of interest.
